# ATHENA: A Personalized Platform to Promote an Active Lifestyle and Wellbeing Based on Physical, Mental and Social Health Primitives

**DOI:** 10.3390/s140509313

**Published:** 2014-05-23

**Authors:** Muhammad Fahim, Muhammad Idris, Rahman Ali, Christopher Nugent, Byeong Kang, Eui-Nam Huh, Sungyoung Lee

**Affiliations:** 1 Ubiquitous Computing Lab, Department of Computer Engineering, Kyung Hee University, Yongin-Si 446-701, South Korea; E-Mails: fahim@oslab.khu.ac.kr (M.F.); idris@oslab.khu.ac.kr (M.I.); rahmanali@oslab.khu.ac.kr (R.A.); 2 School of Computing and Mathematics, University of Ulster, Newtownabbey, Co. Antrim, Northern Ireland BT37 0QB, UK; E-Mail: cd.nugent@ulster.ac.uk; 3 School of Computing and Information Systems, University of Tasmania, Australia; E-Mail: Byeong.Kang@utas.edu.au; 4 Internet Computing and Network Security Lab, Department of Computer Engineering, Kyung Hee University, Yongin-Si 446-701, South Korea; E-Mail: johnhuh@khu.ac.kr

**Keywords:** active lifestyle, wellness services, activity-awareness, personalization

## Abstract

Technology provides ample opportunities for the acquisition and processing of physical, mental and social health primitives. However, several challenges remain for researchers as how to define the relationship between reported physical activities, mood and social interaction to define an active lifestyle. We are conducting a project, ATHENA(activity-awareness for human-engaged wellness applications) to design and integrate the relationship between these basic health primitives to approximate the human lifestyle and real-time recommendations for wellbeing services. Our goal is to develop a system to promote an active lifestyle for individuals and to recommend to them valuable interventions by making comparisons to their past habits. The proposed system processes sensory data through our developed machine learning algorithms inside smart devices and utilizes cloud infrastructure to reduce the cost. We exploit big data infrastructure for massive sensory data storage and fast retrieval for recommendations. Our contributions include the development of a prototype system to promote an active lifestyle and a visual design capable of engaging users in the goal of increasing self-motivation. We believe that our study will impact the design of future ubiquitous wellness applications.

## 1. Introduction

An active lifestyle is the process of enabling technology to improve a person's health and wellbeing in a proactive manner. As such, there is an increasing current desire for societies to engage with technology-based solutions that can prevent conditions from arising and to avoid a deterioration in health-related conditions. Nevertheless, in order to maintain good health, some care is necessary in a number of basic areas. According to the World Health Organization (WHO), health is defined across three primitive attributes; physical, mental and social wellbeing [1]. A strong connection exists between these attributes to maintain a healthy lifestyle [2–4]. For instance, a person with a poor physical health condition can be deemed as being at a high risk of developing a chronic disease, such as diabetes or cardiac-related complications. As a consequence, these factors may affect the person's mental health in terms of inducing depression and social isolation [5]. It is therefore recognized that a balanced combination of maintaining mental and physical health with social interactions can prevent the onset of a chronic disease. In addition to providing a means to manage conditions that may already exist [3,6,7], recently, advances in information communication technologies (ICT), mobile solutions, wearable computing, cloud computing and big data infrastructures are expected to provide cost-effective solutions. It is anticipated that a complete system can accommodate a person's needs from a mental health, physical health and social-wellbeing perspective to provide the necessary levels of required smart care.

Many research studies and assistive devices have been developed to collect personal data to meet the objective of an active lifestyle [8–11]. These technologies contribute a lot toward the adoption in daily routines, reducing cognitive burden and providing a level of comfort. Nevertheless, to date, the developed and evaluated solutions have not fully embraced the importance of physical, mental and social activities under the umbrella of a common platform. For instance, physical activity at a specific time may depend on the mood of the person or the motivation factor behind it. In such a situation, a system capable of providing daily routine interventions, analytics about the health primitives and finding active members related to the subject's interest through social network services can play a significant role in achieving the goal of an active lifestyle.

The aim of the ATHENA research project is to promote active lifestyle and wellbeing by identifying the underline connections between physical, mental and social health primitives. We can relate physical health to exercise routines [8], including sleep, mental health [12] to a person's feelings and emotional states and social-wellbeing [13,14] to the level of outdoor visited places. All these parameters are measured through the usage of pervasive sensing devices and processed both online and offline by our developed machine learning algorithms in order to gain further insight into the current status of the person. In addition, we are maintaining a personal profile to understand user's preferences and interests. On the basis of personal profile parameters, we provide recommendations and interventions to the users. The knock-on effects of such an approach provides reductions in the cost of healthcare provision in addition to promoting an active lifestyle.

The remainder of the paper is organized into different sections. Section 2 briefly describes the related work and their limitations. Section 3 introduces the proposed platform along with individual components required for recognizing the activities, emotions and social interactions. The details of the personal processing and building of the users' profiles are also outlined in this section. Section 4 validates the proposed platform by introducing an active lifestyle case study and provides a detailed discussion. Finally, conclusions are drawn in Section 5.

## 2. Related Work

A recent plethora of work relating to the application of sensor and communication technologies has been successfully developed and deployed to assist users, care givers and healthcare professionals. Grönvall *et al*. [15] provided a holistic picture to understand the non-functional aspects of home-based healthcare technologies. They designed home-based health monitoring practices to better design and integrate them into people's everyday life. They have provided an analysis of the socio-technical complexities through self-monitoring case studies. Fergus *et al*. [16] proposed a framework for the improvement of physical health based on the body area's wireless sensor network and an interactive game. The gathered sensory data is processed and used inside the gaming environment to control the subjects' avatar. An adjustment level mechanism has also been provided in order to change the gaming parameters according to the medical status of the subject. To illustrate the applicability of their approach, they presented a case study of neck physiotherapy.

Titze *et al*. [17] assessed the effects of a sedentary employee's lifestyle and physical activity intervention over change and energy expenditure. For this purpose, they set up six offices of the Swiss federal administration, where a four-month intervention was carried out in each office. Before and after the intervention period, the energy expenditure and the stages of readiness for change during working and leisure time were assessed for lifestyle.

Farooq *et al*. [12] developed a mental activity promotion system that analyzes, quantifies, trains and prescribes based on subject logics and a memorizing capability. They have taken into account the time and space factors for the evaluation of the lifestyle of subjects. Special consideration has also been given to make the system motivational, attractive and fun to use. The system has been successfully deployed in the Bitgeoul senior health town, Gwangju, South Korea.

Li *et al*. [9] highlighted the understanding of personal data, which can be collected through ubiquitous technologies. They identified features like current status, history, goals, discrepancies, context and factors that should be supported in personal informatics tools in order to play an important role. These include current status, history, goals, discrepancies, context and factors. Furthermore, they elaborated that current personal informatics tools are not designed with a sufficient understanding of users' self-reflection requirements.

MIT human dynamics lab [18] introduced the “Reality Commons” platform with the potential capabilities of identifying contexts, recognizing social patterns in daily user activities, inferring relationships and identifying socially significant locations. They logged the time-stamping information about a user's activity, location and proximity to other users. Their applications include ethnographic studies of mobile telephones to measure information access, relationship inferencing and individual and group behavior analysis. They introduced a new dimension of data to inspire the research in a variety of fields. Their platform is limited to the social patterns and sensing social systems. They also highlight that there is much more that needs to be done and that some new dimensions, in the context of user's oriented platform, should be explored.

In previous works, the developed models and frameworks mostly focus either on physical, mental or social health. Nevertheless, a gap exists to model physical, mental and social factors all together for a personal smart care system. To overcome the limitations of existing platforms, there is the need for an alternative state-of-the-art personalized platform to fill in the gap by bridging physical, mental and social interaction in order to promote an active lifestyle in society. Our personalized platform monitors health primitives, suggests interventions and gets feedback from users to provide robust wellness services for an active lifestyle.

## 3. The Proposed Platform

**Figure 1.**
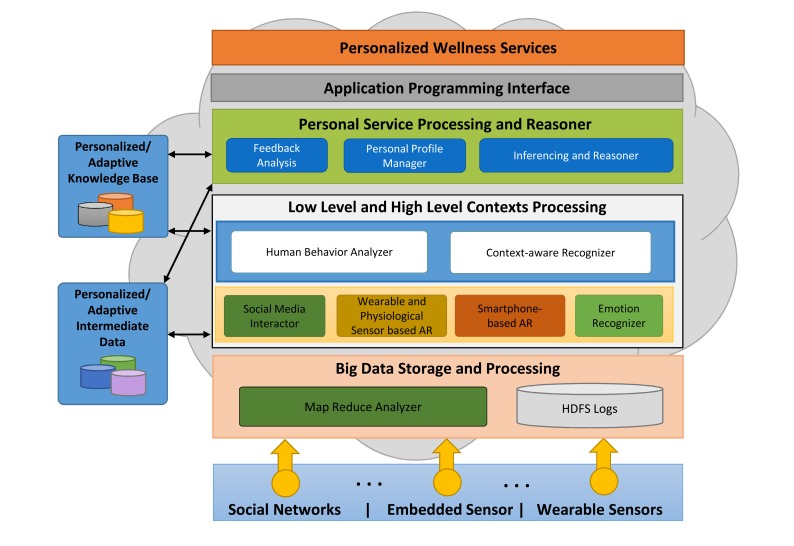
An overview of ATHENA platform.

The proposed platform recognizes the user's activities, emotions and social interaction based on the sensory data originating from the user's smartphone and wearable sensors to support the notion of an active lifestyle. In order to store and process the massive sensory data, big data storage technology and a cloud computing capability are utilized as the backbone of the platform. ATHENA manages the user's profile and the feedback for personalized recommendations on the basis of subject's interests and preferences. Figure 1 shows the enterprise view of the proposed platform. Each component is discussed in detail in the subsequent sections.

### 3.1. Sensor Layer

The sensor layer consists of multimodal sensors, such as embedded sensors in smartphones, wearable sensors and social networks treated as soft sensors. The current generation of smartphones is one of the most feasible and ubiquitous devices, due to their many diverse and powerful embedded sensors. A smartphone includes an accelerometer, magnetometer, gyroscope, proximity sensor, ambient light sensor, GPS and cameras. Furthermore, it is one of the best choices for context recognition, due to its unobtrusive characteristics, high storage capacity and computation, low energy consumption and programmable capabilities. Similarly, wearable sensors are also considered as one of the potential sources to collect user's data. The collected input data is actually the raw logs that are partially structured with respect to sensor categorization. The amount of input data emerging from different heterogeneous sensors is large enough in volume and variety. Therefore, they are stored in Hadoop to exploit the benefits of big data storage.

### 3.2. Big Data Storage and Processing

The partially structured sensory data is stored in the Hadoop distributed file system (HDFS). The structured schema is passed on to the map reduce analysis component to store and retrieve the data. We use Apache Pig scripting language [19] and ZooKeeper [20] technologies to fulfill the data intensive approach in big data technology. The objective of using big data technology for the proposed platform is to provide scalability and to accommodate the bulk amount of user's everyday generated sensory logs. This layer provides the efficient retrieval of the sensory data of the requested user to its upper layer, where low- and high-level context processing is carried out. The retrieved data from HDFS are further stored into the personalized intermediate data for further processing and providing fast access to the sub-modules. Similarly, the personalized and adaptive knowledge bases store and retrieve the information for context-aware recognizer and reasoner components in the forms of ontologies and rule-based storage, respectively.

### 3.3. Low-Level and High-Level Context Processing

In the proposed platform, the user's daily life activities, emotions and social interactions are the most important informative and primitive sources. We process these sources individually and independently by our developed core algorithms. The processed results are stored in adaptive intermediate data for further processing in the personal service processing and reasoner layer. We divide this layer into two levels of processing: low-level and high-level context processing. Low-level context processing provides the primitive information about the activities, emotions and social interactions. While in the second step, we find the high-level contexts and predict human behavior. The details about the technologies developed underneath for the sub-component is described below.

#### 3.3.1. Activity Recognition

Activity recognition (AR) is an important component of our ATHENA platform. This module recognizes the physical activities of the subjects through the use of the embedded sensors of a smartphone. We have developed a comprehensive approach to recognize outdoor activities by utilizing the accelerometer, mic, GPS and WiFi sensor. The solution developed is position free and energy aware, to activate the sensors when required. Activities, such as walking, jogging, riding on a bus or taking a subway and staying in place can be accommodated. We implemented the approach as a smartphone application running on an Android OS and available on the onlinestore as “Action Logger” [21]. Accordingly, we designed and implemented the proposed system, which enabled position-free recognition and was able to recognize activities wherever the smartphone was attached to the body. The evaluations showed that the system works well in real-world environments with an accuracy of 92.43%. These recognized activities are eventually used for inferencing, to provide active lifestyle and wellbeing recommendation services.

#### 3.3.2. Social Media Interactor

Social media technology can play a significant role in reducing the feelings of isolation and levels of depression [22]. Furthermore, social media empowers people to know more about themselves, including their health. For example, research [23] investigated that four out of five users are using the Internet to find personalized healthcare information related to a particular disease and its treatments. In this module, we aim to improve the users' health by utilizing their social interaction in order to suggest to them appropriate lifestyle patterns. Currently, we are processing tweets, trajectory analysis and email interaction. While the details can be found in [24], we also developed a way to post on social media and to get feedback.

#### 3.3.3. Emotion Recognizer

Human emotions have diverse effects on the immune system of a person and, subsequently, have a direct impact on the quality of life. For example, positive emotions contribute to helping fight against the simplest of diseases, such as the common cold [25], recovering from surgery [26] and even protecting against cardiovascular incidents. On the other hand, negative emotions, for example, a high level of depression, may increase the risk of suffering from a stroke [27]. We developed audio-based emotion recognition using the embedded audio sensor of a smartphone. We implemented the approach as a smartphone application running on an Android OS and available on the online store as “Play emotion” [28]. The classification is based on MFCC features and support vector machines (SVM). We performed our experiments on young adults to classify emotions as happy, sad, angry and neutral.

#### 3.3.4. Wearable Sensor-Based AR

Wearable and physiological sensors are one of the most important sources to recognize posture and gesture activities in sports. We used the shimmer sensor [29] under the left or right towel wristband to recognize basketball, badminton and tennis. For instance, after recognizing basketball, we further recognized the gestures such as dribbling, passing, catching and shooting. We extracted time domain features, that is the mean, standard deviation, zero crossing rate for each axis and correlation between axes. We classified activities through a Gaussian mixture model (GMM).

#### 3.3.5. Context-Aware Recognizer

This module works in two phases. In the first phase, it uses mappers and transformers to convert the received low-level context data into a predefined ontological structure. The converted data is forwarded to a context analyzer module for context checking. If the received data contains only an ontological data model, then the context analyzer module applies a match making algorithm to find the match. If the data contains any rule, then the context analyzer extracts the rule and applies a rule-based filtering algorithm. The parser module generates a query on the basis of matching or rules and retrieves the required activity data. Once the pattern of data is matched, it is labeled and forwarded to a decision-making module for a higher-level decision. The decision-making module performs some actions against the suggestions based on the collected contexts.

#### 3.3.6. Human Behavior Analyzer

The understanding of human behavior can prove helpful for supporting an active lifestyle and wellbeing. This module takes the low-level and high-level contexts as input and analyzes a subject's short-term and long-term behavior. It applies the fusion techniques (*i.e.*, horizontal and vertical) to fuse the contextual data to get more sophisticated contextual information. The fusion process is carried out whenever there exists any semantic relationship among the contextual data. Behavior verification is performed to verify the behavior.

### 3.4. Personal Service Processing and Reasoner

The objective of this module is to provide personalized wellbeing services to its users by processing the low-level and high-level context information. Each component is described in more detail in the following sections.

#### 3.4.1. Inferencing and Reasoner

Our proposed inferencing technique is a hybrid approach based on case-based reasoning, rough sets theory and Bayesian decision theory. Initially, our system checks the induction rules generated by the rough set theory and recommends services to the users. As the rough set rules are IF-THEN statements that work on the idea of the exact matching of the parameters of the input service request and the rules already stored in the system's knowledge base, therefore, sometimes, they result in no recommendation, either because of the missing or incomplete parameters in the service request. To reduce this effect, we use a probabilistic model- and case-based reasoning approach for approximating the decision. The case-based approach with the nearest-neighbor technique is used to approximate the top *n* recommendations, and then the Naive Bayes (NB) prediction model is used to validate the decision generated either by the rule-based or case-based approach.

#### 3.4.2. Personal Profile Manager

To provide customized services, personal information, such as gender and age, plays an important role. Once we know about personal information, then one is more likely to adopt the provided recommendation. When new users register to our ATHENA platform, we ask about the subject's preferences in terms of preferred exercise activity, food, music and connected social networks. Once the inference and reasoner module generates the recommendations and interventions, the system sorts them out on the basis of the user's preferences.

#### 3.4.3. Feedback Analysis

To make the platform robust and accurate, a feedback mechanism is introduced to analyze the user's response over the recommended wellness services. The users provide feedback about the provided services through our prompt labeling scheme over smart devices (smartphones and tablets). The details about the prompt labeling scheme are describe in [30]. The component of personal service processing asks the user about the current contexts, as well as the rating of the service. A user can rate the service, provide plain text and share his/her views for the correctness of the service.

### 3.5. Application Programming Interface

This layer provides the information sharing API that can deliver personalized recommendations and wellness services according to the user's selected devices (*i.e.*, smartphone, tablet or web application). The other accountability of this layer is to share subject's routines over social networks to highlight the motivation factors among the network members. Moreover, it exposes the underlying modules to the developers, so that they can mold them for their applications.

### 3.6. Personalized Wellness Services

Web-based analytics, smartphones and tablets are selected as the end nodes to deliver customized wellbeing services. Our analysis emphasizes the relevance of physical, mental and social wellbeing for an active lifestyle to build a personalized platform. On the basis of the user's lifestyle, we are providing the wellness services in the form of exercise, food and social networking recommendations. Finally, sharing the user's life pattern over the social networks with his/her permission promotes an active lifestyle.

## 4. Case Study Scenario and Discussion

Consider the weight management scenario for people, like Mr. Alex Kim. He is a 30-years-old man, who wants to adopt an active lifestyle in his daily routines. He preferred physical exercise such as walking and cycling. His recent weight gain has prompted him to adopt physical activities in daily routines. His daily routines also include socializing with friends and eating fast food during his hangouts. Furthermore, he is also an active user of social networks, like Facebook [31] and KakoStory [32] (*i.e.*, Korean social network) to check-in the restaurants and share his mood and frame of mind. Statistics about his body mass index (BMI) and preferred interactions are summarized in Tables 1 and 2, respectively.

Alex is interested to know about his health primitive statistics, which tells him how much physical activity he did in the previous days, how many calories were consumed during exercise and how much caloric intake, if he provide information about his intake of food. Furthermore, the system should recommend to him a routine plan in terms of daily routines to achieve his active lifestyle goal.

**Figure 2.**
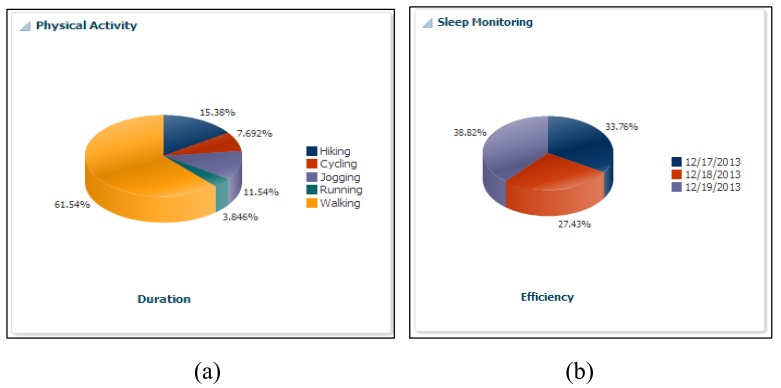
Health primitive physical activity visualization.

**Figure 3.**
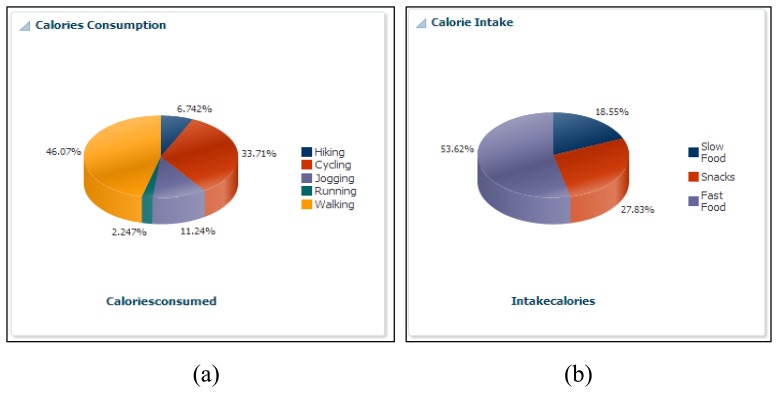
Health primitive calorie visualization.

**Figure 4.**
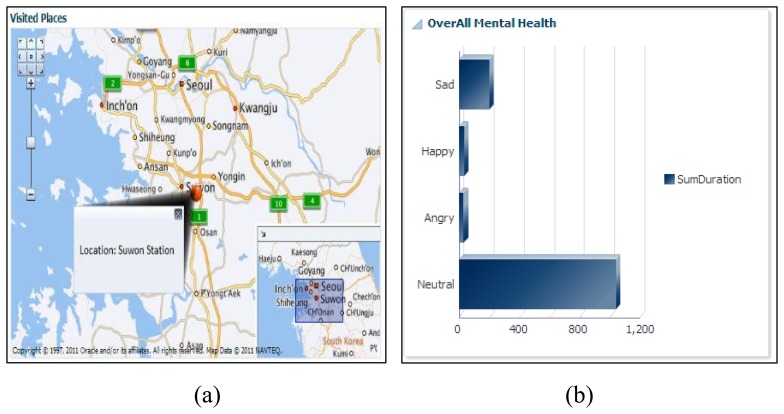
Health primitive social interaction and mental health visualization.

**Figure 5.**
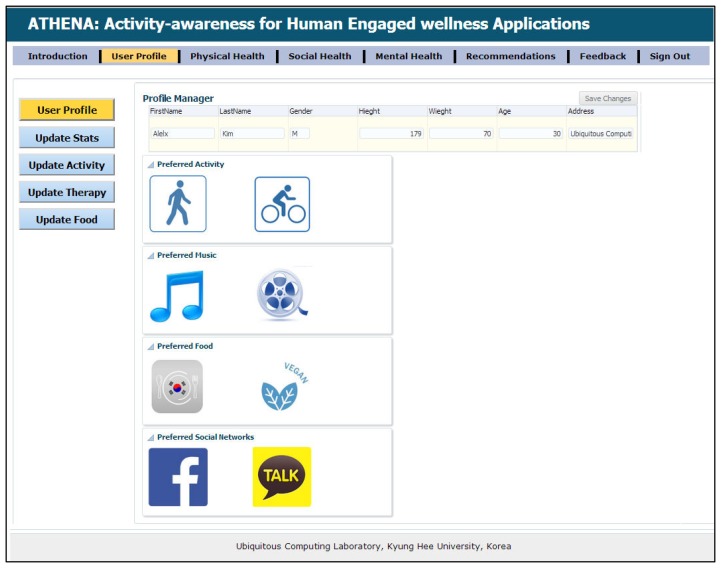
Personal profile manager.

Supporting this scenario means taking over the role of the expert planner and helping the ATHENA platform, which can help him in a truly ubiquitous manner to log his health parameters. In addition, he is free to maintain his diary about daily exercise workouts, burned calories, sleeping routines and food intake calories. Alex downloads our developed application from the Android application store, such as Action Logger [33], Step Counter [34], Human Black Box [35] and Play Emotion [28]. To log his routines and visited location information, our android application has a beta version released. The details are available in [36]. Our ATHENA platform provides web interfaces [37] that give access to visualizing the subject's daily, monthly and weekly physical routines through our graphical widgets. Figures 2, 3 and 4 show the health primitive visualization of Alex. He can maintain his personal profile, change the preferences according to the seasonal change and share his workouts over the social networks with family and friends. The sharing feature of our ATHENA platform has great potential to motivate subjects in terms of appreciation from the social network (in terms of likes) and to promote an active lifestyle. Consequently, it motivates the other members to adopt healthy routines to keep themselves healthier. Figure 5 shows the personal profile management along with the user's preferred physical activity, food and therapy.

**Figure 6.**
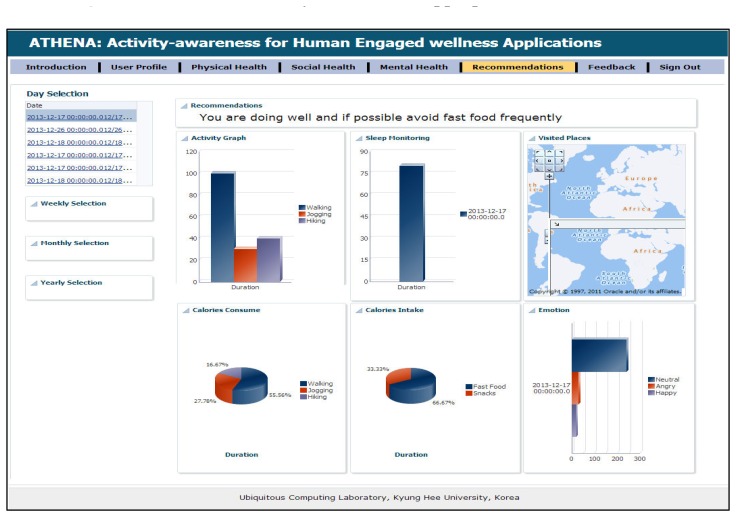
Dashboard of daily routines and appropriate recommendations.

We are also collecting information about age, gender, height and weight to calculate the burned calories, as well as appropriate wellness recommendations. For the evaluation of ATHENA, our feedback mechanism adjusts the recommendations according to the user feedback about the provided recommendations. Figure 6 shows the dashboard of user's daily routines and appropriate recommendations based on his health primitives. Our work shows great potential for promoting an active lifestyle to improve individual health, as well as the population's health.

**Figure 7.**
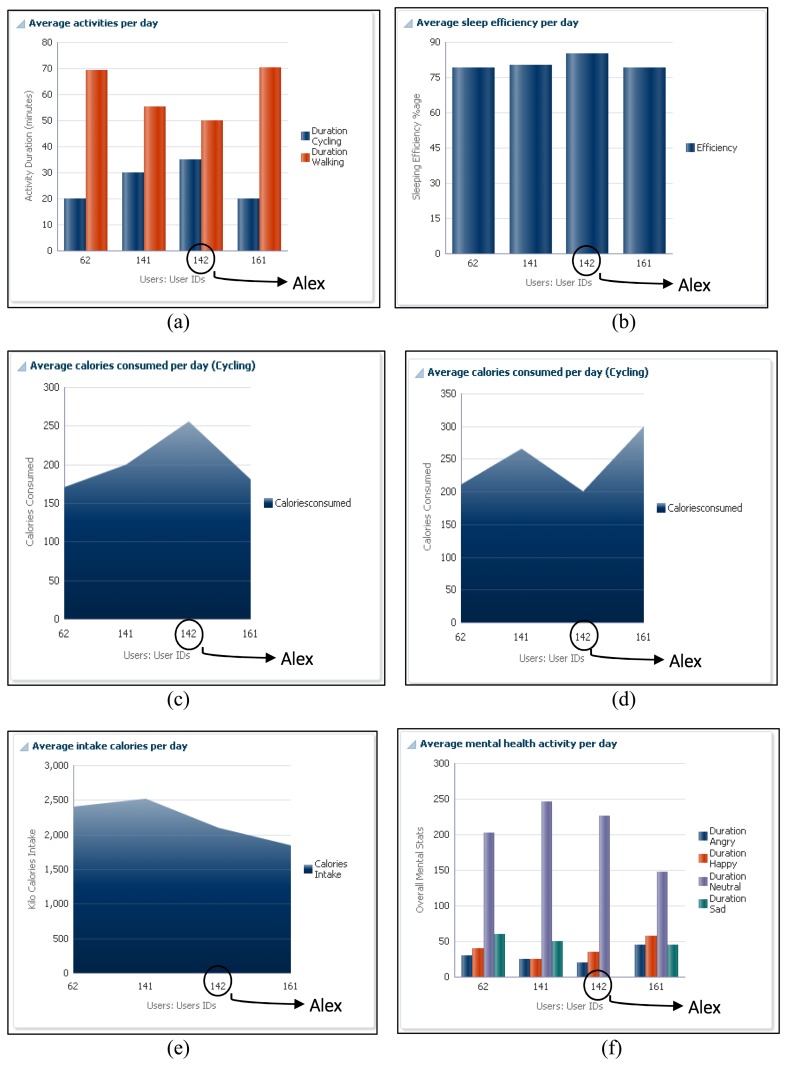
Health primitive comparison with the same group of people (age, height, and weight).

The ATHENA platform also provides a comparative analysis for individuals based on body mass index (BMI) ranges. In order to maintain the privacy of user's, we assign the user identities and group people according to subject interests. In scenario for Alex, we compared his physical activities, consumed calories during exercise, intake of calories in terms of food, sleep efficiency and mental health, with a similar group. In Figure 7a, his cycling activity is more than the other group of persons, while walking is less than the others. Figure 7b shows that sleep efficiency is more than the others. Similarly, in Figure 7c, he consumed more calories as compared to the other group members and performed more cycling *vice versa* (*i.e.*, Figure 7d). Comparing his mental health, it is observed that his average level of happiness is more than the mentioned group of people, and he is less sad.

Plenty of research has been conducted on effective information visualization, but it has not all focused on personal data. Commercial products, such as Nike+ [38], Run Keeper [39] and Sleep as Android [40], can provide assistance to visualize the information. These products are less focused on covering the physical, mental and social interaction health primitives all together. We explore the existing strong connections between the health primitives and describe how existing technology can provide an echo system to promote an active lifestyle. We maintain the user's profiles to deliver personalized wellness recommendations. In addition, providing the current status and their history, people can figure out what goals will be appropriate for them to pursue; for example, maintaining the weight of users' requires keeping a balance in food intake or physical exercise. On the other hand, users' sleep efficiency is decreased, and they feel tired in daily routines. Instead of leaving physical exercise, users can track what kind of food they took and which time they went for exercise. ATHENA can provide recommendations, such as “do not do physical exercise or take coffee X hours before sleeping”. In this way, a user can change his lifestyle while adopting an active lifestyle.

## 5. Conclusions

To promote an active lifestyle, we proposed a personalized platform to provide wellness services to users based on mental, physical and social health. We determined the underlying connections between these health primitives. The proposed platform recognizes the user's activities, emotions and social interaction based on the sensory data originating from the user's smartphone and wearable sensors. In order to store and process the massive sensory data, big data storage technology and a cloud computing capability is utilized as the backbone of the platform. We are managing the user's profile and feedback for personalized recommendations on the basis of subject's interests and preferences. Another important point of this research is to provide services according to the user's selected device (*i.e.*, web interface, smartphone or tablet). Using ATHENA, individuals can perform self-checks for their lifestyle and can also perform self-management. Our future plan is to open our platform for ordinary users and to evaluate its performance. We expect a great impact on promoting an active lifestyle and wellbeing for individuals, as well as for society.

## Figures and Tables

**Table 1.** User's physical statistics.

**Gender**	**Age (Years)**	**Height (cm)**	**Weight (lbs)**
Male	30	179	156.5

**Table 2.** User's preferences and interests.

**Physical Activity**	**Mental Activity**	**Social Activity**	**Preferred Food**
Walking	Music	Facebook	Korean Food
Cycling	Movie	KakoStory	Vegetarian
